# Aptamarker prediction of brain amyloid-β status in cognitively normal individuals at risk for Alzheimer’s disease

**DOI:** 10.1371/journal.pone.0243902

**Published:** 2021-01-04

**Authors:** Gregory Penner, Soizic Lecocq, Anaëlle Chopin, Ximena Vedoya, Simone Lista, Andrea Vergallo, Enrica Cavedo, Francois-Xavier Lejeune, Bruno Dubois, Harald Hampel, the INSIGHT-preAD study group, for the Alzheimer Precision Medicine Initiative (APMI)

**Affiliations:** 1 NeoNeuro SAS, Villejuif, France; 2 Sorbonne University, GRC n° 21, Alzheimer Precision Medicine (APM), AP-HP, Pitié-Salpêtrière Hospital, Paris, France; 3 Brain & Spine Institute (ICM), INSERM U 1127, CNRS UMR 7225, Paris, France; 4 Institute of Memory and Alzheimer's Disease (IM2A), Department of Neurology, Pitié-Salpêtrière Hospital, AP-HP, Paris, France; 5 Qynapse, Paris, France; Consejo Superior de Investigaciones Cientificas, SPAIN

## Abstract

The traditional approach to biomarker discovery for any pathology has been through hypothesis-based research one candidate at a time. The objective of this study was to develop an agnostic approach for the simultaneous screening of plasma for consistent molecular differences between a group of individuals exhibiting a pathology and a group of healthy individuals. To achieve this, we focused on developing a predictive tool based on plasma for the amount of brain amyloid-β deposition as observed in PET scans. The accumulation of brain amyloid-β (Aβ) plaques is a key risk factor for the development of Alzheimer’s disease. A contrast was established between cognitively normal individuals above the age of 70 that differed for the amount of brain amyloid-β observed in PET scans (INSIGHT study group). Positive selection was performed against a pool of plasma from individuals with high brain amyloid and negative selection against a pool of plasma from individuals with low brain amyloid This enriched, selected library was then applied to plasma samples from 11 individuals with high levels of brain amyloid and 11 individuals with low levels of brain Aβ accumulation. Each of these individually selected libraries was then characterized by next generation sequencing, and the relative frequency of 10,000 aptamer sequences that were observed in each selection was screened for ability to explain variation in brain amyloid using sparse partial least squares discriminant analysis. From this analysis a subset of 44 aptamers was defined, and the individual aptamers were synthesized. This subset was applied to plasma samples from 70 cognitively normal individuals all above the age of 70 that differed for brain amyloid deposition. 54 individuals were used as a training set, and 15 as a test set. Three of the 15 individuals in the test set were mis-classified resulting in an overall accuracy of 80% with 86% sensitivity and 75% specificity. The aptamers included in the subset serve directly as biomarkers, thus we have named them Aptamarkers. There are two potential applications of these results: extending the predictive capacity of these aptamers across a broader range of individuals, and/or using the individual aptamers to identify targets through covariance analysis and reverse omics approaches. We are currently expanding applications of the Aptamarker platform to other diseases and target matrices.

## Introduction

Improvement in disease diagnostics is key to developing effective treatments for a variety of pathologies. Identification of biological markers specific to a stage and/or type of pathology is crucial to developing effective treatments. Diseases such as Crohn’s disease, pancreatic cancer, lupus, nonalcoholic steatohepatitis (NASH), and Alzheimer’s disease are all in need of greater understanding of disease pathology and biomarker identification.

In Alzheimer’s disease, the over-accumulation of brain amyloid-β (Aβ) is one of the earliest pathomechanistic alterations characterizing the complexity of AD [1, 2]. In some cases, brain Aβ accumulation may begin to occur twenty to thirty years prior to the onset of symptoms [3]. It is possible to directly observe increased brain Aβ deposition through positron emission tomography (PET) scans, or through analysis of Aβ peptides measured in cerebrospinal fluid (CSF). These analyses are expensive, time-consuming, and invasive, constraining broad-scale application on cognitively normal individuals for clinical trial consideration. However, detecting accumulation of Aβ when it begins to occur gives rise to a potentially more effective therapeutic intervention.

Recent successful achievements with the analysis of Aβ peptide ratios in blood are encouraging as less expensive and minimally invasive means of detecting AD-related pathophysiological dynamics in the preclinical stages of the disease [4–7].

With this in mind, we set out to develop a biomarker-based diagnostic method to screen for molecular differences between cognitively normal individuals above the age of 70 with varying levels of amyloid-β brain deposition based on phenotypic contrasts. Using deep data work to agnostically enable the identification of blood-based biomarkers specific to AD pathology, we apply aptamer library selection, Next Generation Sequencing (NGS), and an analysis of aptamer frequency across individuals to develop an Aptamarker [8] approach to amyloid-β brain deposition prediction.

It would seem that nature chose proteins as the means of enabling antigen detection through antibodies rather than oligonucleotides because the diversity of side chains within twenty amino acids provides higher levels of information potential than the diversity of nucleotides within four nucleotides. With traditional aptamer selection this constraint is overcome through the use of a naïve library of 1E15 random sequences, which is larger than the predicted naïve library of antibodies present in humans (1E6 to 1E7) [9]. The immune system compensates for the small size of the initial random library by refining antibodies that bind to novel antigens through a rapid mutation process. Conceptually though the initial naïve aptamer library is analogous to the antibody repertoire that resides within each individual. The capacity exists to recognize all antigens with high affinity and specificity with the sequences that are present in this library. The key to unlocking this potential is to establish a contrast such that only those aptamers that bind to molecular differences across individuals that are affected by a pathology from those that are not affected by such a pathology. This reduces the problem from identifying all aptamers that bind to everything in plasma, to identifying all aptamers that bind to molecules that are enriched in blood as a function of a pathology.

A primary challenge with aptamer selection is parsing aptamer sequences that bind to a target molecule from those that do not. In traditional SELEX approaches, this is achieved by immobilizing the target and washing away aptamer sequences that do not bind. However, target immobilization compromises the capacity of selection, as the act of complexing a target molecule to a support structure can result in a shape or complex that would not exist outside of the immobilization system. We invented FRELEX (Patent no: US 10,415,034 B2) to enable the partitioning of bound aptamers from unbound aptamers in a way that does not rely on target immobilization, allowing us to select aptamers against all of the complexes found among the molecular constituents of plasma in their native state. In this study, we are able to screen for aptamers that optimally bind to biomarkers in plasma that are enriched in amyloid-β brain deposition biomarkers as they would naturally appear *in vivo* without a need to immobilize these targets first.

Using these approaches we have developed the concept of the Aptamaker, wherein aptamer binding within a system–in this case, pooled serum from individuals with brain amyloid-β deposition–can act directly as biomarkers themselves. The relative frequency of the Aptamarker is a surrogate for the relative frequency of the biomarker it binds to.

This study represents the first application of the Aptamarker platform to the examination of human blood samples and development of a potentially effective screening and diagnostic tool for AD that can replace the expensive and invasive PET scan/CSF analysis.

## Materials and methods

### Preparation of DNA library and primers

The ssDNA library for selection was composed of a 40-mer random region flanked by two constant regions for primer hybridization 5’ AACTACATGGTATGTGGTGAACT (N40) GACGTACAATGTACCCTATAGTG 3’ (TriLink Biotechnologies, San Diego, CA, USA). Primers used for amplification were purchased from Integrated DNA Technologies.

### The INSIGHT-preAD study group

We designed a large-scale mono-centric research program using a cohort of Subjective Memory Complainers (SMC) recruited from the “INveStIGation of AlzHeimer’s PredicTors in Subjective Memory Complainers” (INSIGHT-preAD) study, a French academic university-based cohort which is part of the Alzheimer Precision Medicine Initiative (APMI) and its Cohort Program (APMI-CP) [10–13]. Participants were enrolled at the Institute of Memory and Alzheimer’s disease (*Institut de la Mémoire et de la Maladie d’Alzheimer*, IM2A) at the Pitié-Salpêtrière University Hospital in Paris, France. The main objective of the INSIGHT-preAD study is to explore the earliest preclinical stages of AD through intermediate to later stages until progression to conversion to first cognitive symptoms, using comprehensive clinical parameters and biomarkers associated with cognitive decline. Written informed consent was provided by all participants. The study was approved by the by the INSIGHT-preAD Scientific Committee in October 2017 as Project 48 by the INSIGHT-preAD Scientific Committee. Members of this committee at that time were, Bruno Dubois, Hovagim Bakardjian, Habib Benali, Olivier Colliot, Marie-Odile Habert, Harald Hampel, Foudil Lamari, Fanny Mochel, Marie-Claude Potier, Michel Thiebaut de Schotten). This approval process was conducted in accord with the Helsinki Declaration of 1975.

The INSIGHT-preAD study includes 318 cognitively and physically normal Caucasian individuals, recruited from the community in the wider Paris area, aged 70 to 85, with SMC. Aβ-PET investigation was performed at the baseline visit, as a mandatory inclusion criterion. Thus, all individuals enrolled into the study have SMC and are stratified as either positive or negative for cerebral Aβ deposition. At the point of the study inclusion, comprehensive baseline data were collected, namely demographic and clinical data, and *APOE* genotype. Exclusion criteria were: a history of neurological or psychiatric diseases, including depressive disorders. The study was conducted in accordance with the tenets of the Declaration of Helsinki of 1975 and approved by the local Institutional Review Board at the participating center. All participants or their representatives gave written informed consent for use of their clinical data for research purposes.

### PET data acquisition and processing

All Florbetapir-PET scans are acquired in a single session on a Philips Gemini GXL CT-PET scanner 50 (± 5) minutes after injection of approximately 370 MBq (333–407 MBq) of Florbetapir. PET acquisition consists of 3 x 5 minutes frames, a 128 x 128 acquisition matrix and a voxel size of 2 x 2 x 2 mm^3^. Images are then reconstructed using iterative line-of-response row-action maximum likelihood algorithm (LOR-RAMLA) (10 iterations), with a smooth post-reconstruction filter. All corrections (attenuation, scatter, and random coincidence) are integrated in the reconstruction. Lastly, frames are realigned, averaged and quality-checked by the CATI team. CATI is a French neuroimaging platform funded by the French Plan Alzheimer (available at http://cati-neuroimaging.com).

Reconstructed PET images are analyzed with a pipeline developed by CATI, according to a method previously described. The mean activity in the pons and whole cerebellum regions was used as reference for individual voxel normalization in the partial volume effect corrected images. Standard uptake value ratios (SUVR) were calculated for each of 12 cortical regions of interest (cingulum posterior right and left, cingulum anterior right and left, frontal superior right and left, parietal inferior right and left, precuneus right and left, temporal mid right and left), as well as the global average SUVR. For Aβ-PET data from the CATI pipeline the threshold identified to categorize our cohort in Aβ-PET positive or Aβ-PET negative was 0.7918 [14, 15].

The characteristics of the 70 individuals included in this study are provided in the table below (Table 1).

**Table 1:** Characteristics of 70 SMC individuals analyzed in this study.

	Negative	Positive
Amyloid status	31	39
Average age	77.4 ± 3.1	77.4 ± 3.7
Sex	16F	21F
APO ε status		
E2/E3	5	1
E3/E3	22	20
E3/E4	3	16
E4/E4	1	2

### Pre-treatment of plasma for enriched library development

Depletion on plasma used for aptamer library selection was performed with the Albumin/IgG Removal Kit (Pierce) according to the manufacturer's recommendations. Aliquots (10 μL) of each of the pooled samples were placed in spin columns containing 80 μL of gel slurry. Each column was capped, vortexed, and incubated for 30 minutes at room temperature on an orbital shaker. The column was then centrifuged at 1,000 × g for 2 minutes, and the filtrate was retained and aliquoted for subset analysis.

### Aptamer library selection on plasma

NeoNeuro SAS (France) was kindly permitted to use the FRELEX selection platform (Patent no: US 10,415,034 B2) for this study by NeoVentures Biotechnology Inc. (Canada) For the selection of aptamer libraries. FRELEX requires the preparation of an immobilization field consisting of a gold chip coated with thiolated random 8 base pair DNA oligonucleotides. The 8-mer thiolated random oligonucleotides were dissolved in 50 μL of phosphate buffer saline (PBS) (8.0 mM Na2PO4, 1.4 mM KH2PO4, 136 mM NaCl, 2.7 mM KCl, pH 7.4) at a concentration of 10 μM. This solution was incubated at room temperature (RT) for 1 hour on gold surface chip, dimensions 7 x 10 x 0.3 mm (Xantec, Germany). The chip was then air-dried and 50 μL of a solution containing thiol terminated polyethylene glycol (SH-PEG) molecules and incubated for 30 min at RT with gentle shaking. This step blocks any remaining gold surface that is not covered with 8mers. SH-PEG was subsequently added a second time for 16 hours. After that, the SH-PEG solution was removed from the chip and the functionalized gold chip surface was washed with de-ionised water and air-dried.

In the first step of FRELEX employed in this study, 10^16^ sequences from the random aptamer library described previously were snap cooled by heating the library to 95°C for 10 min followed by immediate immersion in ice bath. These single stranded (ss) DNA sequences were incubated with the functionalized immobilization field (gold chip with 8mers) in 50 μL of Selection Buffer (20 mM Tris, 120 mM NaCl, 5 mM MgCl_2_, 5 mM KCl, pH 7.5) for 30 min at RT. The remaining solution was removed and discarded. This removes sequences that have too much secondary structure to enable binding to the 8mers on the surface. The immobilization field was washed twice with 50 μL of 10X TE buffer and the remaining bound oligonucleotides were eluted and recovered with two incubations of 15 min each 1 mL of Selection Buffer at 95°C. These elutions were pooled and purified using the GeneJET PCR Purification Kit (Thermo Fisher Scientific, Germany) as described by the manufacturer and eluted with 25 μL of de-ionised water.

This aptamer library selected for capacity to bind to 8mers was then used for positive selection with a pool of plasma from six individuals that exhibited high levels of Aβ brain lesions (> 0.79 SUVR) in a total volume of 50 μL 1X Selection Buffer. This solution was incubated for 15 min then incubated with the immobilization field for 15 minutes at RT. The remaining solution was recovered carefully and stored in a fresh tube. The immobilization field was washed twice with 50 μL of 1X selection buffer with each wash being collected and pooled with the solution removed in the first step. This solution contains sequences that did not bind to the immobilization field, presumably because they are bound to some moieties within the plasma instead. This pooled solution was purified as described for the phase I step, eluted in 400 μL and subjected to PCR amplification for an appropriate number of cycles to create a clear band of approximately 5 ng of amplified DNA.

After the first round of selection, PCR was used to amplify the selected ssDNA into double stranded DNA (dsDNA) for an appropriate number of cycles to create a clear band of approximately 5 ng of amplified DNA. For the PCR amplification the 3’ primer was extended beyond homology with the library (in the 3’ direction) with the addition of a sequence corresponding to a T7 RNA polymerase promoter. The amplified dsDNA was used as a template for in vitro transcription to obtain an antisense RNA library. This antisense RNA library was treated with DNase I and purified with RNeasy MinElute cleanup (Qiagen). It was then used as the template in a reverse transcription reaction with reverse transcriptase to obtain sense strand cDNA. The resulting cDNA-RNA mixture was treated with RNase H according to manufacturer instructions. The sense strand cDNA was purified and used as a library for the next round of selection.

From the second to the 10th selection round, selection was performed in the same manner with the exception that a pool of plasma was prepared from six individuals from the INSIGHT-preAD study group that exhibited Aβ brain lesions at a lower SUVr than the threshold of 0.79 as defined by the INSIGHT-preAD study group. Aliquots of this healthy plasma pool was added in the negative selection phase (phase I of FRELEX), where we select for aptamers that exhibit the capacity to bind to the immobilization field. This process was maintained from selection round 2 to selection round 10.

### Characterization of a subset of meaningful aptamers

After ten rounds of selection, aliquots of the enriched library were applied for a single round of positive selection against individual plasma samples of 22 SMC individuals (11 SMC-A+ and 11 SMC-A–). These plasma samples were not depleted for albumin or IgG. Each of these 22 selected libraries of aptamers was characterized by NGS analysis. The relative frequencies of the top 10,000 sequences in terms of copy number were correlated with Aβ brain lesion status using sparse partial least squares–discriminant analysis (sPLS-DA). Based on this analysis a subset of 44 aptamers was defined as sufficient to obtain sensitivity and specificity of 1.0 on the 22 individuals with PLS-DA analysis and cross validation.

### Application of subset of aptamers in a predictive test

The 44 aptamers identified as meaningful from NGS analysis were divided into three subsets of 13, 10 and 23 aptamers each with two aptamers being in common between the first two subsets. Specific primers targeted for the 3’ end of the random region were defined for each aptamer. These primers were validated for their capacity to specifically amplify the targeted aptamer in the presence of the other aptamers in the subset. All subsets were applied in a single round of FRELEX against non-depleted plasma from 70 SMC individuals from the INSIGHT-preAD cohort. The first two subsets were characterized by qPCR analysis on an Mx3000P thermocycler (Stratagene, AH Diagnostics, Aarhus, Denmark) with 10X Sybr green master mix according to the manufacturer's instructions (Bio-Rad). 20 μL reactions included 5 μL of template from selection and 250 nM PCR primers. PCR was performed as follows: 95°C for 2 min; 30 cycles [95°C for 10 s; 62°C for 15 s; 72°C for 30 s]. The last subset was characterized on a BioRad CFC instrument in an identical manner except that reactions were performed for 35 cycles instead of 30.

Cq values were calculated on the basis of the derivative of each amplification cycle exceeding six standard deviations of the average derivative from PCR cycles 3 to 10. PCR efficiency was calculated by determining the slope of the increase in fluorescence from the Cq value to the Cq value characterized as nearest to the maximum derivative across cycles. Cq values from the BioRad instrument were calculated by the internal BioRad software.

### Data analysis

All statistical analyses were performed using the R software program (version 3.4.4) [16]. To assess the predictive ability of the Cq values, we conducted a sparse partial least squares discriminant analysis (sPLS-DA) [15] as implemented in sPLS-DA function of the R package mixOmics^22^. The use of sPLS-DA is motivated by its dimension reduction effectiveness through the construction of a small set of orthogonal components (latent variables) summarizing the manifest Cq values. All of the Aptamarker values and the ratios between them are considered variables. We only computed the ratios in one direction. This still means however that the number of variables far exceeds the number of samples analyzed and as such increases the risk of spurious relationships between variables and variance for Aβ brain deposition. The sparse component of this package enables us to reduce the number of variables and dimensions in which variance is considered to mitigate this problem.

A predictive model was built based on a random sample of 54 individuals from the 70 individuals analyzed. One individual sample was excluded from the analysis as an outlier. This left an additional 15 individual samples as a test set. For data analysis, ratios of all Cq values from the 54 samples were determined and added to the Cq values alone as a combined dataset. This data set was normalized to zero mean and standard deviation of unity for each Cq value or Cq value ratio. This normalized matrix was then used as explanatory variables in sPLS-DA to account for the Aβ brain lesion status, categorized as negative “neg” or positive “pos” based on a SUVr threshold of 0.79.

In addition, a cross-validation analysis was performed with sPLS-DA with a manual k-folds analysis. Alternative sets of 54 training samples and 15 test samples were chosen 5 times. An sPLS-DA model was created with each of the iterations of the training set and applied to the test set. We allowed the sPLS-DA to normalize the training set to mean = 0 and standard deviation = - 1. We used the mean and the standard deviation from these training sets to apply the same normalization to the test sets. The predicted values for the test set were evaluated for whether they predicted low or high amyloid by subtracting the mean of the negative samples in the training set from the observed value, and dividing by the standard deviation of the negative samples in the training set, and likewise for the positive samples in the training set. The value that was the closest to zero arising from this evaluation was used to establish the low or high amyloid prediction.

The overall number of samples was too small for us to evaluate the effect of genotype or sex on the model. A difficulty with sPLS-DA model fitting is that when the model is unbalanced in the sense that one class of individuals outweighs the other class considerably, the model tends to favour the more dominant class at the expense of predicting the less dominant class appropriately. With partitions for either sex or ApoE genotype the model failed because of unbalanced training sets. Both sex and genotype will be considered fully in our current work with a larger data set.

## Results

**Fig 1:**
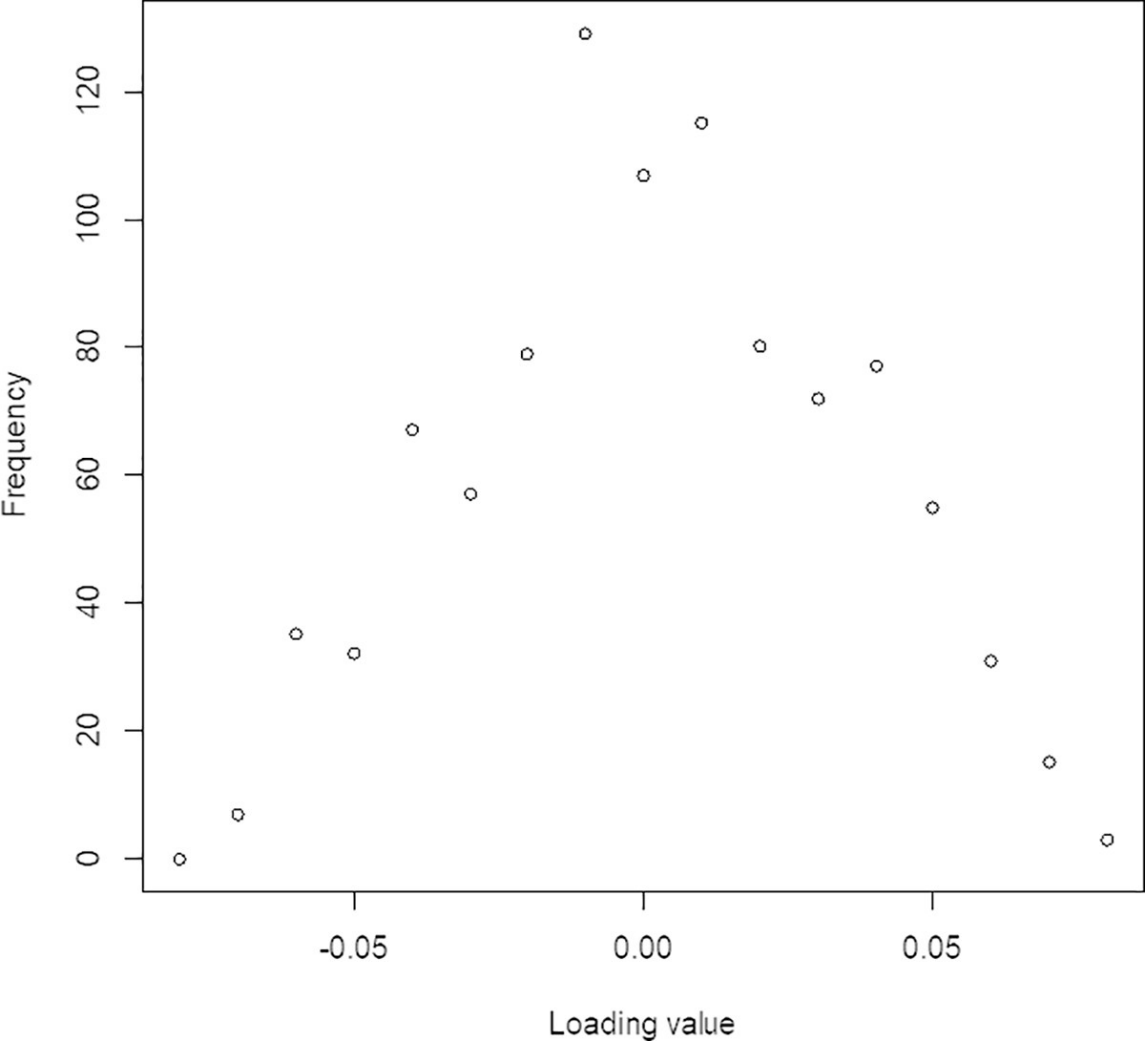
Frequency distribution of loading values. Frequency distribution of loading for 2116 retained variables on 54 sample training set.

**Fig 2:**
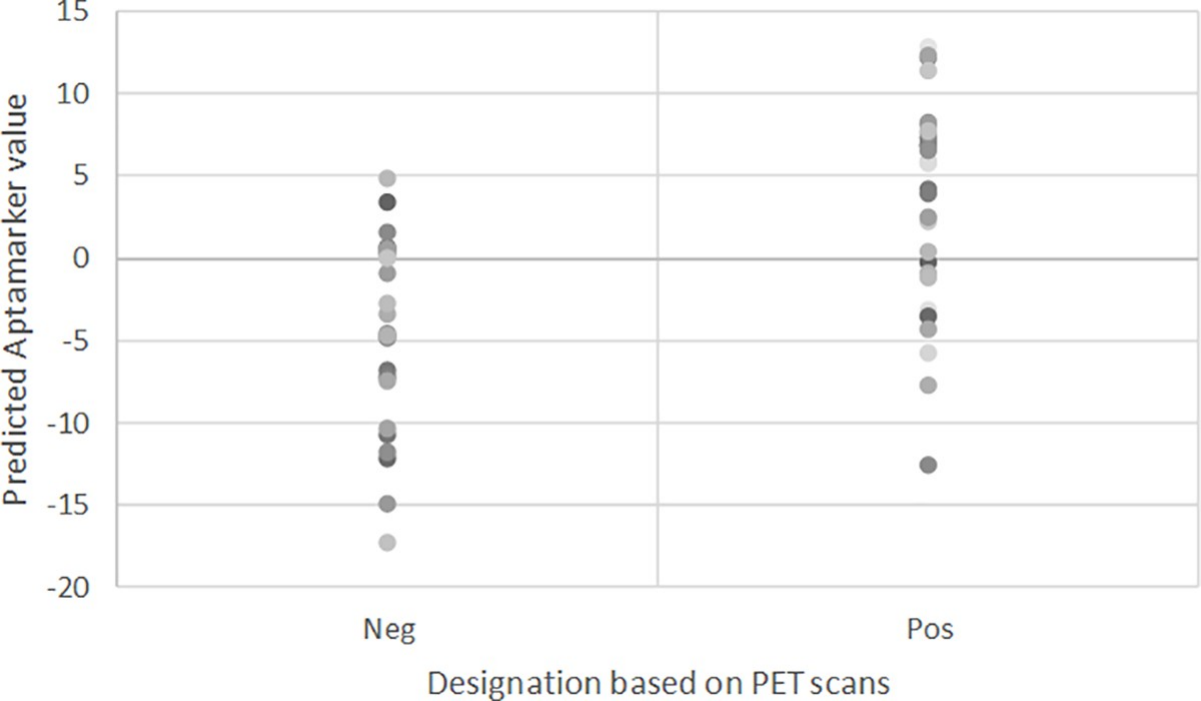
Distribution of values. Distribution of values assigned to 54 training samples based on mathematical model of Aptamarkers.

The sPLS-DA analysis of the 54 training samples resulted in the retention of 961 variables, all explaining variance in one principle component. The loading values of these 961 variables were distributed as shown in Fig 1. The majority of the loading values had values that deviated from 0 by less than 0.01. The distribution of the training samples based on a SUVR threshold of 0.79 is shown in Fig 2. The average predicted model result for the low brain Aβ deposition (SUVR < 0.79) was -4.76 while the average for high brain Aβ deposition (SUVR > 0.79) was +3.53.

We applied this mathematical model to the Aptamarker results obtained with 15 samples that were not part of the initial training set with the following confusion matrix result.

                   Aptamarker negative    Aptamarker positive

Pet scan negative                   6                    2

Pet scan positive                   1                    6

**Fig 3:**
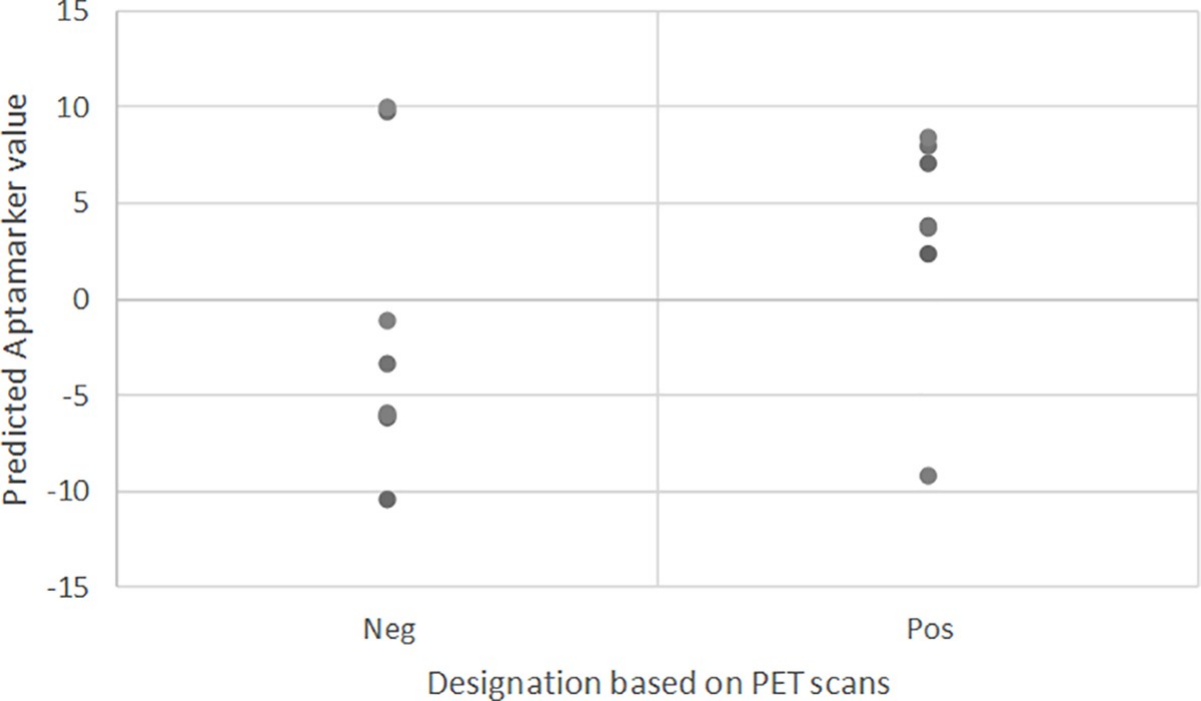
Distribution of values assigned. Distribution of values assigned to 15 test samples based on mathematical model of Aptamarkers.

**Fig 4:**
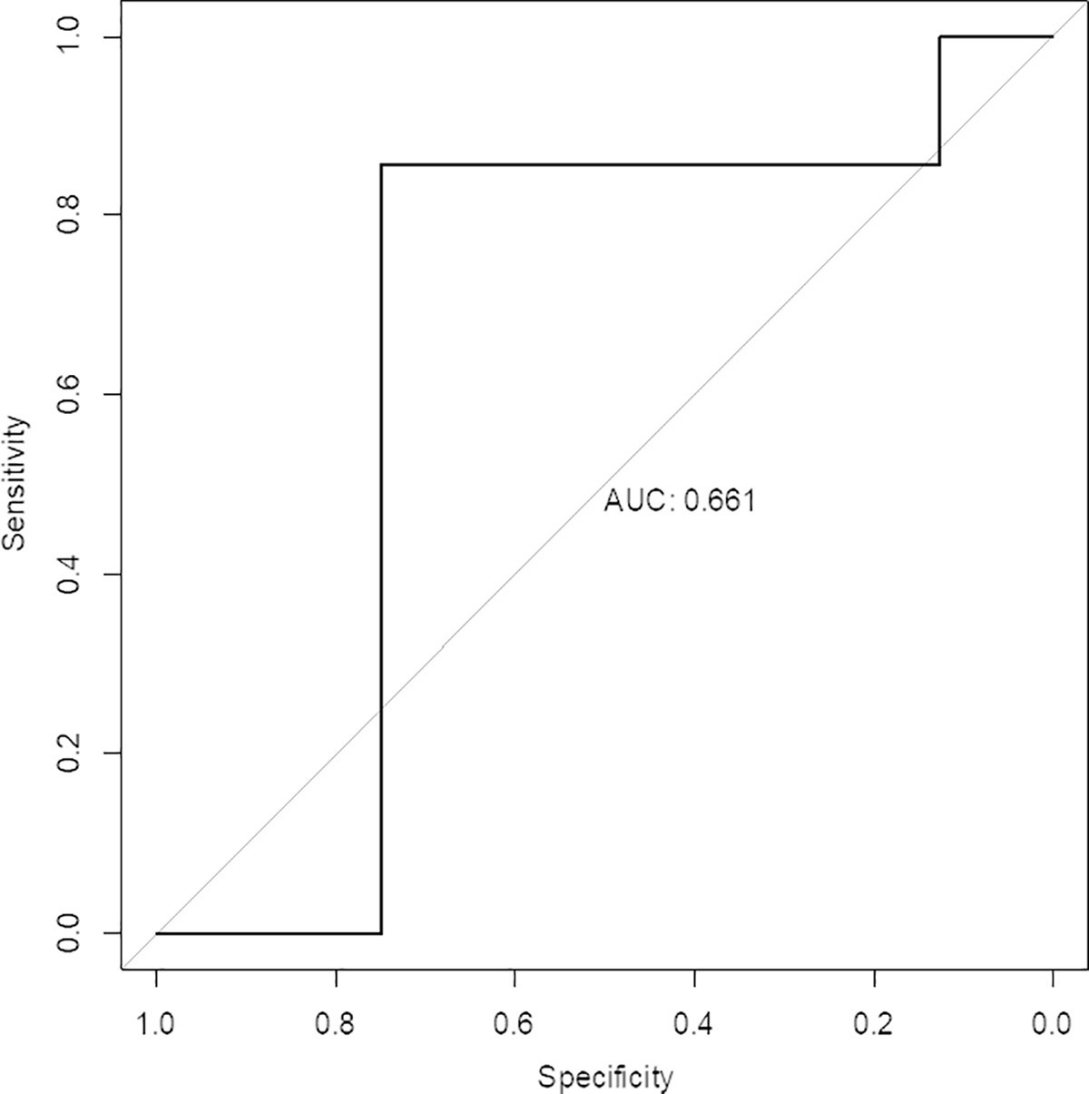
ROC curve. ROC curve for 15 test samples based on Aptamarker predictions.

This results in a sensitivity of 85%, a specificity of 75% and an overall accuracy of 80%. The distribution of the individual 15 test samples based on their predicted values is provided in Fig 3. The ROC curve for these 15 samples is provided in Fig 4. The ROC curve is provided here as a reference, it is clear that it is difficult to obtain robust results and hence high area under the curve (AUC) values with a test set of only 15 samples as variation in the threshold leads rapidly to skewing of sample designation distribution. In either direction, the proportion of negative or positive samples changes more rapidly than would be the case with more samples.

We determined that by including only those variables that exhibited loading values greater than 0.04 or less than -0.04 this confusion matrix was maintained. This reduces the number of variables necessary within the prediction to 245. The inclusion of an individual Aptamarker in a ratio value varied from a minimum of three ratios to a maximum of 37.

The average result from the cross-validation analysis was 70% for sensitivity, 59% for specificity and 65% for accuracy. These values demonstrate that the model as it stands is not sufficiently robust for commercial development. There is a need to improve this model through analysis of more samples.

## Discussion

By training our enriched aptamer library against a random sample of 54 individuals from the original INSIGHT-preAD group pool, and applying it to the remaining relevant 15 individuals, we observed a predictive accuracy of 80%, with 86% sensitivity, and 75% specificity for brain amyloid deposition. The cross-validation exercise resulted in lower predictive accuracy (65%), lower sensitivity (70%) and lower specificity (59%). These values demonstrate that the interpretation of the Aptamarker patterns across the individuals can be used to explain a portion of the variation for brain amyloid deposition on the basis of aptamer binding to biomarkers in plasma. We have designated the overall process as the Aptamarkers platform.

To further these results, we are looking to integrate this approach with the results obtained using traditional core pathophysiological biomarkers for AD diagnosis across the same samples: low CSF Aβ_1–42_ concentrations and elevated CSF total tau (t-tau) or hyperphosphorylated tau (p-tau) concentrations or positivity to Aβ-PET (*i*.*e*., high retention of Aβ radiotracer). This will demonstrate whether the variance in the trait that we are explaining using the Aptamarkers platform is identical or whether this strategy represents an added value to the variance obtained by the established core biomarkers.

The concept of using aptamer frequency following a single round of selection as a characterization of the concentration of the targets that aptamers bind to and the subsequent use of aptamers directly as biomarkers is an entirely new concept. The investigation reported herein resulted in a predictive capacity that is similar to that achieved in plasma with Aβ peptide ratios. This is sufficiently encouraging for us to expand this research with increased sample quantities.

Additionally, we are developing mathematical models to assess the efficacy of the aptamer library selection and to further refine the way aptamers are chosen for the third step in the Aptamarker platform.

The evaluation of the second step of the platform–characterization of candidate aptamers through a single round of selection and NGS analysis–is prone to an over-fitting error, given that the number of variables (sequence frequency of aptamers) far outnumbers the number of samples. Different aptamers are chosen as meaningful arising from this step depending on the iteration of the model building software (different random seeds). We are also working on the development of a rigorous process that will enable the identification of meaningful sequences from this data set that recur most frequently. We are building a statistical model to support this aspect of the analysis.

In terms of developing a comprehensive diagnostic tool for AD, as well as other diseases, on an individual level, the Aptamarkers platform approach hold promise. The Aptamarkers platform allows the unbiased assessment of differences in plasma samples independent from the type of the biological molecule [17]. As such, this platform is assessing differences across all possible existing epitopes, a dimension we refer to as “epitopiome”.

The Aptamarkers platform can be represented as a trade for increased depth of analysis (rapid development and characterization of many more potential biomarkers) in exchange for a loss of information regarding the biological nature of the biomarkers. From a diagnostic viewpoint, the nature of the specific biomarker and/or its potential role in the pathophysiology of the disease is not necessary. It is only required to demonstrate that the biomarker provides a statistically relevant diagnosis of the disease. It is entirely feasible to use the Aptamarkers platform as a tool to discover the molecular entities that they bind to by immobilizing each known Aptamarker sequence in an affinity column and passing plasma through it. Given the broad spectrum of potential targets, including proteins, peptides, oligonucleotides, metabolites, and combinations of these molecules, this use of aptamers to discover the identity of the actual biomarkers is not trivial.

A key concern regarding the application of this platform the subject of measurement is unknown in that we do not know what the aptamers are binding to. This concern can be addressed in several ways:

1. The existing relationship between a biomarker and a disease state does not need to be known in order to be useful. The advent of genomic analysis has led to the correlation of genetic variation in proteins of unknown function with the propensity to develop a disease. These correlations are necessarily included in predictive models.
2. The most important concern for a clinician is to correctly diagnose a disease state. Knowledge that a biomarker may be related to a pathophysiological basis for such a state may be of scientific comfort but in practical terms in terms of prescribing treatments is not as important as the statistical correlation between diagnosis, treatment choice and treatment outcome.
3. There are biomarkers that are routinely used in healthcare where the association with the disease is either unknown or tentative at best. For example, with Alzheimer’s disease the using Apo E genetic status as a predictor for the potential to develop the disease. Pathophysiological hypotheses to support the strong statistical correlation are currently being built and tested, but this was driven by the statistical correlation, not the evident relationship with the pathology.
4. It is possible to use the Aptamarkers that we discover to identify what they bind to. The primary difficulty in this approach is the technical constraints involved in identifying complexes between metabolites and mis-folded or non-trypsin-digested peptides, not the ability to use aptamers to pull out specifically bound molecules. We postulate that a more effective approach to identifying binding will be through covariance analysis in association with hypothesis.

The identification of Aptamarkers is not trivial, however their application once identified is straightforward. The process only involves a single positive FRELEX selection on a gold chip, followed by characterization with the Aptamarkers platform through qPCR analysis. This is simple to perform, does not require expensive equipment, and the results are highly reliable (CV < 5%). The platform as it has been developed could be automated and scaled, making it a potentially valuable diagnostic resource.

## Conclusions

Considerable development work is required before commercialization of this approach can be considered. We consider the predictive capacity demonstrated herein for this test as encouraging for analysis of a larger number of samples, further refinement and improvement of the test. The significant correlation between Aβ deposition across the twelve samples predicted correctly was also encouraging given that all training of mathematical models was based on binary separation of low and high amyloid. The observation of such a significant relationship to actual Aβ deposition levels was unexpected and is encouraging in terms of the Aptamarkers describing aspects of the pathophysiology of the disease.

The Aptamarker platform is intriguing because of the agnostic nature of the aptamer selection. This approach allows screening of all possible molecules in blood, proteins, metabolites, non-coding RNA and complexes formed among these molecules for possible relationship to the pathophysiology or pathophysiologies underlying not only an early risk factor for Alzheimer’s disease such Aβ brain deposition, but the pathophysiology of any disease. Efforts are currently underway within our laboratory to extend this application beyond Alzheimer’s disease into other types of diseases.
